# Perfectionism, Body Satisfaction and Dieting in Athletes: The Role of Gender and Sport Type

**DOI:** 10.3390/sports7080181

**Published:** 2019-07-24

**Authors:** Katarina Prnjak, Ivan Jukic, James J. Tufano

**Affiliations:** 1 Department of Psychology, Faculty of Humanities and Social Sciences, University of Zagreb, 10000 Zagreb, Croatia; 2 Sport Performance Research Institute New Zealand (SPRINZ), Auckland University of Technology, Auckland 0632, New Zealand; 3 Department of Physiology and Biochemistry, Faculty of Physical Education and Sport, Charles University, 16000 Prague, Czech Republic

**Keywords:** eating disorders, sport, perfectionistic strivings, perfectionistic concerns, body image

## Abstract

Athletes are often at a greater risk for disordered eating development due to their perfectionistic tendencies, as well as physical performance- and appearance-related demands of various sports in which they compete. Therefore, the purpose of this study was to examine the possibility of independent contributions of perfectionism and body satisfaction on dieting behaviour among male and female athletes. Two-hundred-eighty (192 male; 88 female) athletes provided their answers on the Eating Attitudes Test 26 (EAT-26), Positive and Negative Perfectionism Scale (PANPS) and modified Body Image Satisfaction Scale from Body Image and Body Change Inventory. No gender or sport type differences were observed in dieting behaviour and body satisfaction was the only significant predictor of dieting for female athletes. Mediation analysis demonstrated that body satisfaction is a mediator between both adaptive and maladaptive perfectionism and dieting. These findings emphasize the important role that body satisfaction has in disordered eating development in female athletes.

## 1. Introduction

An upsurge of scientific interest in the area of disordered eating among athletes has been observed, with more qualitative methodologies focusing on how athletes experience this condition [1]. Disordered eating is shown to have a higher prevalence among athletes than general population, even reaching 45% in female athletes [2] and this condition is especially emphasized in sports where weight controlling behaviour is expected, such as martial arts, running, synchronized swimming and gymnastics [3,4]. Moreover, patterns related to eating disorders might be difficult to detect among some athletes [5], which is a problem because eating disorder consequences are oftentimes far-reaching and could impair metabolism, bone health, cardiovascular health and mental health [6]. In addition, a decrease in food intake frequently leads to lower energy availability among athletes, a state that causes hormonal alterations, menstrual irregularities and impaired bone health, which consequently has a negative impact on sport performance as well [7]. 

Examination of athletes’ personality characteristics should fulfil the gaps in the knowledge regarding eating disorder-related outcomes [8]. For example, elevated perfectionism is often observed among those with eating disorder symptoms and is considered to be multidimensional [9], consisting of adaptive (or healthy; perfectionistic strivings) and maladaptive (or unhealthy; perfectionistic concerns) facets [9,10]. In the sport context, adaptive perfectionism was associated with greater training performance [11] and lower levels of training distress [12], while maladaptive perfectionism seems to be a risk factor for exercise dependence [13]. Additionally, Gotwals, Stroeber, Dunn and Stoll [14] noted that perfectionistic strivings in athletes are primarily adaptive, occasionally neutral and rarely maladaptive, when controlling for perfectionistic concerns. However, although adaptive perfectionistic strivings are usually considered essential for the greater sport achievements [15], Flett and Hewitt [16] emphasized that interpreting perfectionistic strivings as adaptive after controlling for perfectionistic concerns might not be a veracious approach in a way that athletes rarely exhibit only perfectionistic strivings without also being highly self-evaluative. A recent meta-analysis by Limburg, Watson, Hagger and Egan [17] showed that both dimensions of perfectionism are often associated with various psychopathological conditions. Likewise, both adaptive and maladaptive perfectionism appear to be elevated in individuals with eating disorder [10]. Furthermore, perfectionism, achievement motivation and competitiveness were addressed as vulnerabilities for disordered eating development in competitive female athletes [18]. Perfectionism was also related to dieting and bulimia symptoms among female college athletes [19] and was shown to be the greatest risk factor for disordered eating among female athletes by Hopkinson and Lock [20]. It is possible that perfectionists, who often set very high standards and struggle to cope even with small failures, are more likely to develop eating disorder once enrolled in a competitive athletic environment [8]. Also, this relationship seems to be highlighted among female athletes rather than males and Somasundaram and Burgess [21] showed that sport type might moderate the relationship between perfectionism dimensions and disordered eating among female athletes. Therefore, the role of adaptive perfectionism might be ambiguous and further examination of its contribution to disordered eating in both female and male athletes would be useful. 

A core symptom of eating disorders is body dissatisfaction [22] but this relationship seems to be less straightforward in athletic population [23]. Exposure to high standards and constant evaluation of physical appearance in some sports can lead to negative body image if these standards are not met [24]. For example, female collegiate athletes symptomatic for an eating disorder reported more perceived pressure to be thin than their asymptomatic peers [25]. This is most commonly noticed in so-called “aesthetic” sports, such as gymnastics, dancing, figure skating [26] and competitive bodybuilding where increased preoccupation with weight and shape, along with body dissatisfaction has been reported in the literature [27]. Collectively, these studies indicate that being dissatisfied with one’s own body represents a vulnerability for the development of unhealthy eating habits in female athletes but little is known about this relationship among males. Furthermore, body dissatisfaction could be the factor through which athletes who are perfectionists develop eating disturbances or athletes could experience symptoms of disordered eating due to perfectionistic tendencies solely. Previous studies have proven the mediating effect of body satisfaction between perfectionism facets and unhealthy eating behaviour [28,29]. However, not always were the athletes of both genders included or the role of sport type examined and recent findings provide support for the fact that eating disorders are common but in research often neglected among men [30]. 

Therefore, it would be important to comprehend associations between constructs related to unhealthy eating patterns and what triggers these issues in order to assure better eating disorder prevention among athletes, which would lead to greater health- and performance-related outcomes. Finally, due to dissenting findings in the area of perfectionism research, it would be beneficial to further clarify its relationship with both adaptive and maladaptive outcomes in athletic population. Therefore, the aim of this study was to examine whether adaptive and maladaptive perfectionism predict disordered eating symptoms among female and male athletes of team and individual sport and whether body satisfaction can change the significance of this relationship as a mediator between perfectionism and disordered eating. 

## 2. Materials and Methods

### 2.1. Participants 

Two-hundred-eighty (192 male; 88 female) athletes aged from 18 to 29 years (M = 21.03, SD = 2.18) were recruited at the Faculty of Kinesiology. A larger sample was recruited (*N* = 581) with the help of Faculty staff; however only those respondents who self-identified as athletes currently competing in their respective sport were retained for the analyses. This sub-sample consisted of exclusively Caucasian participants, who on average reported to be engaged in specific sport for 11.59 years (SD = 4.15). Around 57% of participants competed in team sports (e.g., soccer, basketball), 14.7% in fighting sports, 7.6% in aesthetic sports (e.g., gymnastics, figure skating, etc.) and 20.9% in other individual sports, such as athletics and swimming. For the latter analyses, these sport types were categorised as either team sport or individual sport (fighting, aesthetic and other individual sports combined) to assure sufficient and approximately the same number of participants in each category for the analyses to be performed. Forty-seven percent of female athletes participated in team sports, while 60.9% team sport players were observed among males.

### 2.2. Measures

#### 2.2.1. Sociodemographic Questionnaire 

Participants provided answers on questions regarding their gender, age, year of college, type of sport and duration of sport participation. These sociodemographic questions were listed at the end of the printed, hardcopy version of the questionnaires. 

#### 2.2.2. Perfectionism 

Positive and Negative Perfectionism Scale (PANPS; [31]) is a 40-item measure of both adaptive aspect (e.g., I like the acclaim I get for an outstanding performance) and maladaptive aspects of perfectionism (e.g., When I start something I feel anxious that I might fail). Participants provided their answers on 5-point Likert-type scale, from *strongly disagree* to *strongly agree*. The Cronbach Alpha coefficient of internal consistency for adaptive and maladaptive perfectionism was 0.83 and 0.81, respectively [32]. Internal consistencies for adaptive perfectionism and maladaptive perfectionism subscales in the present study were 0.78 and 0.84, respectively.

#### 2.2.3. Body Satisfaction 

*Body image satisfaction scale* from *Body image and body change inventory* [33] usually consists of 10 items (e.g., How satisfied are you with your body shape?) that measure satisfaction with each body part. This questionnaire was modified and validated (α = 0.92) by Erceg Jugovic [34] and this modification was used in the present study. Four items related to satisfaction with height, muscle firmness, waist and bottom were added, which makes total of 14 items in the scale. Participants answer on 5-point Likert-type scale from *very dissatisfied* to *very satisfied*. Internal consistency of the original scale was shown to be 0.92 [33]. In the current study, the internal consistency measured as Cronbach Alpha was 0.90 for the modified version of the scale. 

#### 2.2.4. Disordered Eating 

The Eating Attitudes Test-26 (EAT-26; [35]) consists of 26 items and is one of the most widely used measures of disordered eating behaviours and attitudes. Participants provided their answers on 6-point Likert-type scale, from *never* to *always*. Three factors were defined in EAT-26: dieting (e.g., I avoid foods with sugar in them), bulimia and food preoccupation (e.g., I vomit after I have eaten) and oral control (e.g., I cut my food into small pieces). Internal consistencies of the three mentioned factors on a sample of anorexia patients were α = 0.90, 0.84 and 0.83, respectively [35]. In the current study, coefficients of internal consistency for the three factors were 0.75, 0.59 and 0.28, respectively. Due to low internal consistency, only first subscale (dieting) was kept as a measurement of disorder eating symptoms in this study.

### 2.3. Procedure 

Participants were recruited in collaboration with Faculty of Kinesiology staff members and data was collected during course lectures. General instructions were read out loud, after which attendants could either agree to take part and skip the participating. Those who agreed were provided with the printed versions of questionnaires by the investigators (who were not engaged in academic courses). The questionnaires were randomly organised to prevent any order effects. The completion of all questionnaires lasted between 10 and 15 min and afterwards the fulfilled questionnaires were returned to the researchers. The response rate after administration of questionnaires was higher than 95%. All procedures were carried out in accordance with the Declaration of Helsinki and were approved by the University of Zagreb Human Research Ethics Committee. All participants gave written informed consent prior to participating. Participants did not receive any compensation for their participation in the present study. 

### 2.4. Statistical Analyses

Normality of data distributions was tested using Shapiro-Wilk’s test of normality. Mann-Whitney U test was carried out to compare gender differences in dieting due to non-normal distribution of data. Spearman correlation analyses were performed for variables with skewedly distributed data but separately for males and females, as well as hierarchical regression analyses. Mediation analyses were computed on overall sample to test body satisfaction as a mediator between (1) adaptive perfectionism and dieting; and (2) maladaptive perfectionism and dieting. Significance was accepted at *p* < 0.05. All statistical analyses were performed using the software package SPSS (IBM SPSS version 24.0, Chicago, IL, USA) with a Process Macro add-on [36] for the purposes of mediation analyses.

## 3. Results

### 3.1. Preliminary Analyses

Only scores on maladaptive perfectionism were normally distributed according to Shapiro-Wilk’s test. Dieting scores were hence converted into z-values to obtain normal distribution of data. The linearity assumption for the main variables was not violated. For each analysis missing values were handled with listwise method and thus 233 participants were retained in the study. 

### 3.2. Descriptive Statistics and Correlational Analysis

Means and standard deviations were calculated for the main variables separately for female and male athletes (Table 1). Results of Mann-Whitney U test showed no significant difference (U = 7374.50, *p* = 0.653) between female and male participants. In females, body satisfaction significantly correlated with both perfectionism dimensions and dieting, while in males only significant correlation between body satisfaction and maladaptive perfectionism was observed (Table 1). The two facets of perfectionism were not significantly correlated in females nor males (*p* > 0.05). 

### 3.3. Hierarchical Regression Analysis

The results of the hierarchical regression analysis are demonstrated in Table S1 (see Supplement 1). In the first step, age, sport type and duration of sport participation were introduced but no significant contribution was made (*p* > 0.05). In the second model, two facets of perfectionism were included but none of them contributed significantly (*p* > 0.05). Finally, in the third model, body satisfaction was added and this variable independently contributed to the explanation of dieting (*p* < 0.05). Overall, the set of these predictors explained 18.10% of dieting variance. However, for the male athletes, none of the predictors contributed independently (*p* > 0.05) to the explanation of dieting and only 5% of dieting variance was explained using these predictors. 

### 3.4. Mediation Analysis 

**Figure 1:**
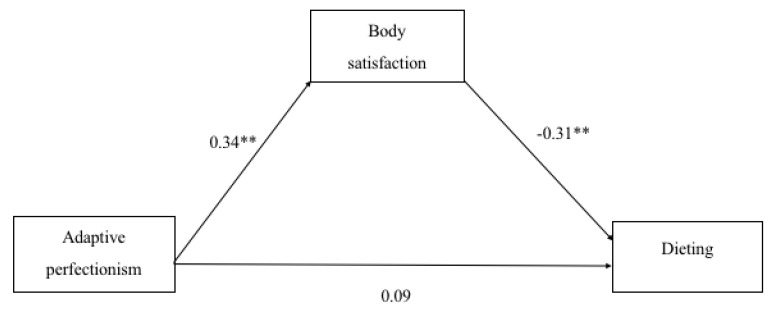
Beta coefficients as indicators of the association between adaptive perfectionism, body satisfaction and dieting in female participants (n = 79). * *p* < 0.05. ** *p* < 0.01.

**Figure 2:**
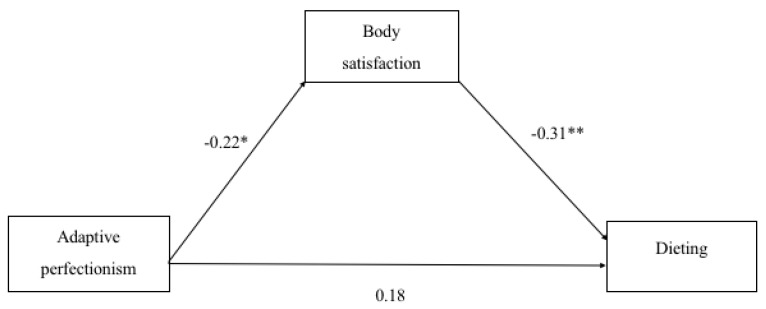
Beta coefficients as indicators of the association between maladaptive perfectionism, body satisfaction and dieting in female participants (*n* = 79). * *p* < 0.05. ** *p* < 0.01.

According to the meditation analyses, beta coefficient was statistically significant (*p* < 0.05) for total effects in case of maladaptive perfectionism but not for adaptive perfectionism (Table 2). Bootstrapping method in mediation analysis between adaptive perfectionism and dieting and maladaptive perfectionism and dieting, resulted in indirect effect whose confidence intervals do not include value zero and thus confirm their significance. These relationships are demonstrated in Figure 1 and Figure 2.

## 4. Discussion

Findings in the present study indicate that perfectionism and body satisfaction explain dieting among female athletes to some extent, as evidenced by the results of the mediation analysis which yielded a significant indirect effect. Nevertheless, due to confidence intervals being very close to value zero, these effects might not be large enough to be considered as important for explaining dieting behaviour among females. For the male athletes, none of the predictors contributed to the understanding of their dieting behaviour. In addition, age, sport type and duration of sport participation did not contribute to dieting behaviour in case of male and female participants in the present study. 

Male and female athletes of the current study did not differ in the prevalence of dieting behaviour. Generally, the prevalence of eating disorders is higher in females than in males and among athletes compared to non-athletes [2]. Precisely, Bratland-Sanda and Sundgot-Borgen [2] reported a prevalence range of 0–19% in male and 6–45% in female athletes. The findings of the present study are thus somewhat unexpected since women generally exhibit a higher risk for developing an eating disorder, mostly due to expectations from the society and thin-idealization [37]. Nordin-Bates et al. [38], in their longitudinal study, reported that the percentage of male and female dancers who scored above the cut-off in disordered eating attitudes changed between time points of data collection. Cross-sectional studies, like the present one, however, cannot compare time points due to obvious limitation and one-point data collection may not yield stable findings. With this in mind, prevalence of male and female disordered eating symptoms might have differed if we collected data longitudinally. 

Adaptive and maladaptive perfectionism were associated solely with body satisfaction among female athletes in the present study but not with dieting behaviour directly. Although Ferrand and Brunet [29] also reported no significant contribution of adaptive perfectionism to eating disorder symptoms among male cyclists, Somasundaram and Burgess [21] showed that evaluative concerns (maladaptive perfectionism) have higher association with disordered eating than strivings (adaptive perfectionism) in both female athletes and non-athletes. Further, Hopkinson and Lock [20] reported that professional athletes exhibited more eating disorder symptoms than recreational athletes, mostly because of elevated general perfectionism than intensity of training. To our knowledge, in the area of disordered eating, no research has yet demonstrated the potentially beneficial role of adaptive perfectionism dimension among athletes. Hence, the results of the current study are thus in contradiction with previous studies as we found adaptive perfectionism to be positively associated with body satisfaction, which is negatively linked to dieting. Perhaps the adaptive perfectionism dimension captured in present study is highly determined by the self-report tool being used, as different perfectionism questionnaires focus on different aspect of this trait. In fact, adaptive perfectionism measured by PANPS might be more representative of positive feelings associated with achieved outcomes, not strivings per se [39]. However, inconsistent findings warrant more scientific interest in this area. 

Satisfaction with one’s body was shown to be the largest independent predictor of dieting in the present study and their relationship was negative. This relationship between body satisfaction and disordered eating is not entirely straightforward in the case of the athletes as female college athletes actually reported higher body satisfaction than non-athletes but also elevated perfectionism related to unhealthy eating attitudes, which was accentuated among athletes in judged sports [19]. However, Ferrand, Magnan, Rouveix and Filaire [40] showed that dissatisfaction with body weight was positively associated with both self-oriented perfectionism and disordered eating in synchronized swimmers and operated as a mediator between these concepts. Therefore, it would be important to further establish the relationship between perfectionism dimensions and body satisfaction in the area of disordered eating but also take into a consideration the context in which athletes perceive their body [23]. With that being said, athletes recruited for the current study were on-going comprehensive education about physical activity (including psychological aspects related to it) which potentially increased their awareness and consequently might have served as a protective factor against development of disordered eating symptoms. However, possible influence of participants’ education was not examined in the present study.

Sport type did not predict dieting behaviour in the current study, which is somewhat unexpected finding since sport type was shown to play a significant role in motivation for reshaping body and development of body dissatisfaction [41]. However, when measuring disordered eating as an outcome, Haase [42] showed that athletes of individual sports experienced a higher level of disordered eating symptoms than team sport athletes, whilst in another study, sport type did not play a role in disordered eating among male and female athletes [20]. Therefore, the role of sport type seems to be inconsistently supported in the literature and our results agree with those in the study by Hopkinson and Lock [20]. Perhaps using more sport categories in the present study, such as aesthetic and fighting sports, would have demonstrated somewhat different findings than the ones we observed.

Several limitations of the present study should be noted. The data was based on self-reported measures, which often contain a certain amount of dishonesty and socially desirable answers. Cross-sectional design of this study seriously limits the causal inference between perfectionism, body satisfaction and dieting. Furthermore, the convenient sample in the present study consisted of athletes who were also enrolled in a university program (kinesiology students) which might limit the generalizability to other athletes who might not be familiar with potential issues related to perfectionism and body satisfaction. In addition, potential restrictions came from the measurement instruments used for data collection since some researchers (e.g., [39]) have suggested that PANPS should not be used as a measure of perfectionism due to a questionable factor validity of the “positive perfectionism” subscale. Furthermore, EAT-26 was shown to have a low positive predictive value due to the rarity of targeted conditions [43] while two out of three EAT-26 subscales demonstrated low internal consistency and omitting them while investigating disordered eating may not be the best approach. Finally, the number of female participants was notably lower than males, which might have affected some of the analysis were gender-split was included. 

## 5. Practical Applications 

Coaches sometimes have difficulties in recognizing disordered eating symptoms in individual athletes, especially if their body composition is in accordance with specific sport expectations and performance is not impaired [44]. Because of that and considering how far-reaching eating disorder impacts can be, there is a necessity for implementation of proper assessments of eating disorder in athletes using an objective, reliable and validated tools. This should serve as a first step in identifying potential risk factors often manifested in the interaction of athletes’ personality traits and social environment in which sport preparation and performance occurs. Hence, the cooperation between coaches, sport nutritionists, clinical psychologists and athletes themselves is essential in preventing potential eating disorder development among athletes. In addition, raising awareness about body image issues that follow male athletic involvement and education about different presentation of male eating disorders among professionals dealing with athletes is warranted. 

## 6. Conclusions

No associations were observed between perfectionism, body satisfaction and dieting for the male athletes that participated in this study. For the female athletes, body satisfaction appeared as the most important factor in explaining dieting behaviour and additional analyses showed that body satisfaction may operate as mediator between both adaptive and maladaptive perfectionism but these effects are rather small. Findings presented here indicate that dieting, as a symptom of disordered eating, could have different underlying factors in case of female and male athletes, although the level of exhibiting this behaviour may not differ between the two genders.

## Figures and Tables

**Table 1:** Spearman’s correlation coefficients, means and standards deviations of the variables in this study for female (*n* = 79) and male (*n* = 165) participants.

**Females (*n* = 79)**				
**Variable**	**(1)**	**(2)**	**(3)**	**(4)**
(1) Dieting	–			
(2) Adaptive perfectionism	−0.04	–		
(3) Maladaptive perfectionism	0.263 *	0.08	–	
(4) Body satisfaction	−0.308 **	0.254 *	−0.22 *	–
*M*	5.02	77.39	54.22	50.43
*SD*	4.93	8.74	9.65	8.17
**Males (*n* = 165)**				
**Variable**	**(1)**	**(2)**	**(3)**	**(4)**
(1) Dieting	–			
(2) Adaptive perfectionism	−0.06	–		
(3) Maladaptive perfectionism	0.04	0.14	–	
(4) Body satisfaction	−0.03	0.10	−0.27 **	–
*M*	5.04	75.51	54.49	52.95
*SD*	4.60	8.62	10.70	9.22

Note. * *p* < 0.05. ** *p* < 0.01.

**Table 2:** Results of the two mediation analyses between: adaptive perfectionism and dieting; and maladaptive perfectionism and dieting for female participants (*n* = 79).

Predictors	Coefficient	SE	t	Bootstrap SE	Bootstrap 95% CI
Adaptive perfectionism					
Total effect	0.005	0.008	0.678		
Direct effect	0.009	0.008	1.107		
Indirect effect	−0.003			0.002	[−0.008−0.001]
Maladaptive perfectionism					
Total effect	0.013	0.006	2.154 *		
Direct effect	0.011	0.006	1.657		
Indirect effect	0.003			0.002	[0.001−0.007]

Note. * *p* < 0.05; SE—standard error; CI—confidence interval.

## Supplementary Materials

The following are available online at https://www.mdpi.com/2075-4663/7/8/181/s1, Table S1: Results of hierarchical regression analysis with dieting as the criterion variable for female (n = 79) and male (n = 165) participants.
