# Supplementary material for: Perfectionism, Body Satisfaction and Dieting in Athletes: The Role of Gender and Sport Type
Source: Sports (Basel). 2019 Jul 24;7(8):181. doi: 10.3390/sports7080181 (PMC6723820; doi:10.3390/sports7080181)
Supplement: Supplementary file 1 [file sports-07-00181-s001.pdf]

sports MDPI

Supplementary file 1

Table S1. Results of hierarchical regression analysis with dieting as the criterion variable for female (n = 79) and male (n = 165) participants.

**Females (n = 79)**									
**Variable**	**Model 1**			**Model 2**			**Model 3**		
	B	*SE B*	*β*	*B*	*SE B*	*β*	*B*	*SE B*	*β*
Age	0.07	0.06	0.15	0.06	0.05	0.14	0.04	0.05	0.09
Sport type	0.09	0.10	0.11	0.07	0.10	0.09	0.09	0.09	0.11
Sport participation	−0.03	0.03	−0.13	−0.03	0.03	−0.13	−0.03	0.03	−0.12
Adaptive perfectionism				−0.00	0.02	−0.02	0.03	0.05	0.09
Maladaptive perfectionism				0.03	0.01	0.23	0.02	0.01	0.14
Body satisfaction							−0.04	0.01	−0.32*
R^2		0.05			0.10			0.18	
F for ΔR^2		1.23			1.52			2.48	
**Males (n=165)**									
**Variable**	**Model 1**			**Model 2**			**Model 3**		
	B	*SE B*	*β*	*B*	*SE B*	*β*	*B*	*SE B*	*β*
Age	0.04	0.04	0.09	0.04	0.04	0.09	0.05	0.04	0.10
Sport type	0.09	0.06	0.13	0.10	0.06	0.156	0.12	0.06	0.18
Sport participation	0.00	0.02	0.00	0.00	0.02	0.00	0.01	0.02	0.02
Adaptive perfectionism				0.01	0.01	0.08	0.01	0.01	0.10
Maladaptive perfectionism				0.01	0.01	0.08	0.01	0.01	0.05
Body satisfaction							−0.01	0.01	−0.11
R^2		0.03			0.04			0.05	
F for ΔR^2		1.45			1.25			1.35	

Note. SE – standard error. *$p < 0.05$.

© 2019 by the authors. Submitted for possible open access publication under the terms and conditions of the Creative Commons Attribution (CC BY) license (http://creativecommons.org/licenses/by/4.0/).
